# Waiting, strange: transplant recipient experience, medical time and queer/crip temporalities

**DOI:** 10.1136/medhum-2021-012141

**Published:** 2021-05-28

**Authors:** Sara Wasson

**Affiliations:** Department of English and Creative Writing, Lancaster University, Lancaster, UK

**Keywords:** English literature, literary theory, literature and medicine, narrative medicine, medical humanities

## Abstract

People who receive a ‘solid’ organ transplant from a deceased person may experience imaginative challenges in making sense of how the transfer impacts their own past and future, as shown in existing scholarship. Building on such work, this article considers how the temporalities of medical encounter itself may also become temporally ambiguous, posing representational challenges both pre-transplantation and post-transplantation. The dominant narrative of transplant in transplantation journals and hospital communications, both clinical and patient-facing, presents surgery as a healing moment, yet the recipient’s experience of hospital, pharmacology and daily self-monitoring may be disorienting in multiple ways which resist conventional conceptions of medical temporalities of cure. Examining memoirs by Robert Pensack and Richard McCann, this article suggests the transplant temporalities may be fruitfully approached through scholarship of ‘queering’ time and ‘crip’ time. While the medical narrative of transplant focuses on the event of transplantation, these texts construct post-transplant time as still profoundly structured by waiting, expectation and suspense, the transformed body less healed than permanently contingent and fragile in different ways. I do not purport to uncover the ‘truth’ of bleak survival hidden within a story of the miraculous. Rather, I am reaching for a critical practice to recognise subtle entanglements of medicalised time, and identify a tension and synthesis between miracle and the chronic, an insight which may also be of service for other critical approaches to memoir of heroic medicine.

[W]hat happens after the miracle?

- Richard McCann, ‘The Resurrectionist’

How might a medical experience blend heroic event and chronic duration, and how might that entanglement be written? Furthermore, what critical reading practices might help us notice their meshing, and why might that noticing be important? This article considers these questions with reference to two memoirs of transplant recipients, receiving ontologically necessary ‘solid’ organs from deceased donors. I will suggest that some memoirs of heroic medical intervention can be characterised by a tension between two kinds of narrative time. On the one hand, the works foreground the temporality of ‘miracle’—an exalted, epiphanic moment when conventional possibilities are exceeded and life is saved and transformed through exceptional means. On the other hand, the works are informed by the chronicity of long-term illness management, the long durations of both presurgical anticipation and postsurgical aftermath. I do not seek to present the latter as a straightforward corrective to the former, puncturing the illusion of restitution story. Rather, I am interested in the way that these two temporalities necessarily overlap within the narration, notably meeting across images which simultaneously evoke both repair and chronic incurability. The analysis may also be of service for other critical approaches to memoirs of heroic medicine.

## Sudden change and slow struggle: temporalities of transplant care

The dominant narrative of transplantation in transplantation journals and hospital communications, both clinical and patient-facing, presents surgery as a healing *event*, while acknowledging that the recipient will need to follow a pharmacological regimen of immunosuppressants and monitoring for organ rejection. When surgery is emphasised as a remarkable healing event, a time-bounded medical feat which blurs boundaries of life and death, it is not surprising that a language of ‘miracle’ has gathered for centuries around the concept of solid organ surgical transplant from deceased donors. According to legend, in the 6th century CE, Saints Cosmas and Damien conducted the first transplant—a leg from a dead man to a living one—and that story was painted for centuries (Barkan 1996; Trethewey 2012, 11). When transplantation became increasingly feasible, miracle continued to be invoked in a secular sense. Recipients, medical practitioners, donor kin and others may use the language of the miraculous to describe tissue transfer. As a small sample, transplant surgeon Thomas Starzl speaks of transplantation as ‘miracle’ (Starzl 2003, 3), surgeon and historian Paul Terasaki declares that ‘Transplantation is the miracle of 20th century medicine’ (Terasaki 1971, 7) and the term features in medical journals such as *British Journal of Surgery, The New England Journal of Medicine* and the *British Medical Bulletin*, including articles titled ‘The miracle of liver transplantation’ (Tzakis 2013), ‘Transplantation: a medical miracle of the twentieth century’ (Morris 2004) and ‘The miracle of face transplantation after 10 years’ (Siemionow 2016). The language of miracle also extends to patient experience: in interviews with transplant recipients, Pera et al identify a consistent theme of ‘living a miracle’ (Pera et al. 2012, 599).

Crucially, this discourse of miracle coexists with a rather different—but no less celebratory—conception of medicalised time: that of progress arc, both in the sense of advances in medical innovation and the progress of individual healing. Such discourse tends to invite a particular narrative structure, linear and restorative: a lapse from wellness into illness, and then a return to the state of ‘health’, implicitly conceived as a stable and intelligible category. While linear progress and miracle may seem mismatched, the ubiquity of the language of ‘miracle’ in commentaries on transplant can be seen to fit in that the progress arc of heroic medicine requires moments of exalted striving and transcendence, exceeding both prior knowledge and the conventional boundaries of life and death. This was particularly the case in late 20th-century memoir, at which time transplantation had become established, certainly, but was less routinised within popular conception as it arguably is today. What the litany of ‘miracle’ adds, when used about transplant or other boundary-breaking medical work, is a sense that although return to health may occur in an arc of healing progress, that arc requires a particular moment of extraordinary medical intervention.

In patient-facing communications, too, emphasis may understandably be on the surgery as watershed event, after which the emotional challenges of the illness are likely to ebb. Discussing the patient handbook at the hospital where her research occurs, for example, Margrit Shildrick notes that the manual for patients meticulously describes the postsurgical healthcare steps required, but ‘the authorised narrative of the transplant clinic is one in which the rhetoric of hope leaves little room for any exploration or understanding of the more negative affects and emotions that recipients may experience … [w]hat it scarcely addresses … is any of the emotional effects that transplantation might be expected to evoke…. The manual does acknowledge that the indefinite waiting period may be one in which feelings of “fear, impatience, or discouragement—even anger—are normal” … but the absence of any address to the extended post-transplant period effectively situates those feelings as short-term and easily resolved’; (Shildrick 2015, 21–2, 26–27). Such communications tend to frame the process as restitution story as Arthur Frank identifies it, in which medical practitioners restore health and the illness experience is understood within a cultural framework of faith in medical triumph and compliance with its interventions (1995). A large body of work has already shown how restitution story may be inadequate for acknowledging the complex imaginative and conceptual work that recipients may need to undertake after deceased-donor transplant, work which may include imaginatively coming to terms with a new sense of hybridity, and grieving both the donor's death and one’s own changes (Almgren et al. 2017; Crowley-Matoka 2005; Fox and Swazey 1978; Fox and Swazey 1992; Hamdy 2012; Poole et al. 2009; Poole et al. 2014; Sanal 2011; Sharp 2006; Shildrick 2015; Shildrick et al. 2009; Wasson 2020).

Embodiment (both pre-transplant and post-transplant, including ‘normal’ human embodiment) can fruitfully be considered in terms of Gilles Deleuze and Felix Guattari’s concept of ‘assemblage’, a blend of materials tangible and intangible, organic and not, with variable trajectories of deterioration, connecting ‘states of things, bodies, various combinations of bodies, hodgepodges … utterances, modes of expression and whole regimes of signs’ (Deleuze 2007, 177). Margrit Shildrick and Deborah Steinberg show that the concept ‘unravel[s] the conventional notion of the body as a stable, unified and bounded entity, and emphasises the multiple connections that bodies form with other bodies, whether human, animal or machine’ (Shildrick and Steinberg 2015, 16n4). Assemblages materialise in transfer in terms of intersections of human bodies and through the discourses and materialities of waiting lists, self-monitoring and pharmacological regimens. Time, too, is part of these assemblages, a force which changes things. It affects tissues, bodies, subjectivities and systems, such as the artery walls which narrow over time with chronic rejection, or the waiting lists which accumulate in databases. These texts construct both pre-transplant and post-transplant time as structured by waiting, expectation, suspense and dread, the transformed body less ‘healed’ than permanently contingent and fragile in new ways. Tissue transfer from deceased donors may pose particular imaginative pressures on recipient sense of time: both the past and the future may be made strange. Shildrick and McCormack have shown that the recipient may experience their own past as now entangled with another’s history, one which will likely never be known, and their future life as necessarily dependent on the persistent survival of (part of) that other (Shildrick 2015, 21–2; McCormack 2016a, 57). Building on such work, this article considers how the times of *medical intervention,* too, may become strange. Recipients’ experiences of hospital, pharmacology and daily self-monitoring may be disorientating and resist conventional conceptions of temporalities of cure.

Throughout, I will tend to speak of ‘tissue transfer’ rather than the more widely used ‘transplant’. ‘Transplant’ emphasises the event of the recipient’s surgery, while ‘tissue transfer’ more readily encompasses donor surgery and the panoply of mediating practices, parties and structures involved in the movement of tissue (Sharp 2006, 3–4). The temporalities I consider in this article are not ideologically neutral, but freighted with particular conceptions of proper agency and culturally lauded modes of being. To find a language for the specific kinds of temporality I discern in these works, I have turned to scholarship from the fields of queer theory and disability studies, specifically work on ‘queer’ and ‘crip’ time. As I will discuss, these bodies of work notice how alternative kinds of time might inhabit dominant arcs—rogue time, strange pockets, within hegemonic conceptions of either linear healing progress or transcendent moments of ‘cure’. The prose I examine in this article confronts the emotional challenge of difficult waiting, waiting become chronic and permanent including in the aftermath of transfer. In these works, waiting in strain becomes *waiting, strange*, a defamiliarisation from a ‘self’ and from temporal rhythms and aspirations taken for granted by wider culture.

The two memoirs are American, and first published in 1994 and 2000, respectively; as such, their representations are not generalisable across other places or eras. The protagonists are privileged in that they have access to transplantation: in countries without universal healthcare, potential recipients confront a ‘“green screen” of ability to pay’, as Renée Fox and Judith Swazey phrase it (1992, 75), a financial barrier for the surgery and for the subsequent immunosuppression that surgery necessitates. Furthermore, these works are about legal transfer, administered by government-sanctioned apparatus; as such, black or ‘grey’ market transfer, or government-sanctioned organ sale as can apply in Iran, would raise different issues. This specificity is important not least in that the texture of waiting within these works is informed by a sense of being held within a vast, legal, socially-sanctioned apparatus, simultaneously impersonally benevolent and opaque.

The memoirs I will examine invoke miracle, but alongside the epiphanic miraculous they also explore the slow duration of chronic illness, etymologically illness in-and-of time. The latter temporality is not separate from the surgical intervention, but is in fact attendant on and entangled with that intervention. These memoirs are, technically, about ‘recoveries’ in the sense that both transplant recipients survived their transplant and medically speaking did well afterwards, yet both show that process to be inextricably entangled with a non-curative story. These temporalities are tightly interwoven through the works, and as I will show, both works infuse a single, shared image with the meanings of both kinds of time. Waiting becomes relevant to both.

Becoming a transplant recipient begins with waiting: not only for an organ but also, often, waiting to be ill enough to be added to the list, while enduring deterioration and surgical and medical procedures of monitoring and maintenance such as kidney dialysis or cardiac pacemaker insertion. Statistics on waiting times vary within nations, regions and organ types, but it is often a matter of years, if an organ becomes available at all. In the UK, for example, while about half of prospective recipients receive kidney transplants within 1–3 years, some hard-to-match prospective kidney recipients wait for 7 years or more, and in the case of heart transplant, median waiting times vary and are influenced by region and blood group, but can again be years (NHS B&T 2020,NHS B&T 2020, ‘Kidney’ and ‘Cardiothoracic’). NHS Blood and Transplant report that in the last 5 years, 201 patients have died before a heart became available for transplant (NHS B&T 2019). Waiting is affected by inequalities: in some countries it is possible, for example, to be listed multiple times on a transplant register, if supported by a willing insurance provider (Fox and Swazey 1992; UNOS 2020). The list itself is complex and out of patients’ control, maintained by national or regional tissue management authorities such as Organ Procurement Organisations in the USA or NHS Blood and Transplant in the UK, and allocation lists operate with algorithms which themselves have at times been found to inadvertently create inequalities in tissue access (Cecka 2007; Epstein et al. 2000; Johnson et al. 2010; Kierans and Cooper 2011, 136; Su X et al. 2004).

The wait for transplant can be emotionally challenging. In addition to apprehension over one’s own survival and the outcome of the wait, the prospective transplant recipient may also feel guilt over what they are waiting for, if the organ is such that the death of another will be required to procure it (Bunzel et al. 1992, 251–52). There may also be apprehension at the aftermath of surgery, the statistics of survival rates, chronic rejection, and the side effects of immunosuppression, as well as awareness of one’s own progressive decline. During this stage, the uncertainty of the end date of waiting intersects with a shadow form of waiting: waiting for one’s own body to further deteriorate. S. Lochlann Jain’s concept of ‘prognostic time’ is relevant here, in which prognosis ‘demands that we adopt its viewpoint, one in which the conclusion haunts the story itself’ (Jain 2013).

**Figure 1:**
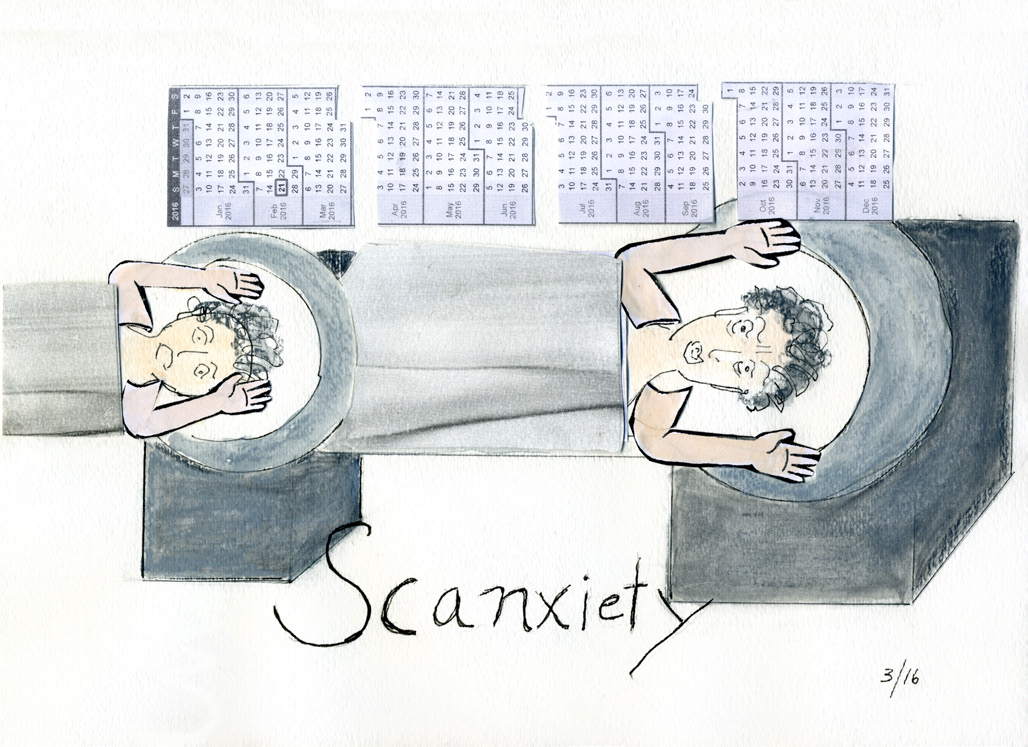
Nancy K. Miller, ‘Scanxiety’, *NancyKMiller.com* (28 July 2016) https://nancykmiller.com/2016/07/cancer-gadfly-scanxiety/ (accessed 25 October 2020). Reproduced with permission of the artist.

Crucially, waiting continues after transplant. Time is still punctuated by monitoring and tests, including daily self-monitoring and sometimes invasive procedures, and the anticipation of forthcoming interventions and rejection. As Robert Pensack and Dwight Williams note, “the reality is that you can feel fine while experiencing life threatening rejection. You need a hospital to tell you what is going on with your heart” (1994, 308). Salient here is Eric Cazdyn’s concept of the ‘new chronic’, in which symptoms are predictably managed, and ‘the utopian desire to cure is displaced by the practical need to manage and stabilise’ (Cazdyn 2012, 6). Cazdyn finds this a deadening temporality that reduces imaginative possibility. The transplant survivor memoirs I discuss do acknowledge a sense of repetitious monitoring and treatment in aftermath, but *un*predictability is very possible and indeed inevitable, given the realities of slow chronic rejection even if acute rejection can be averted. The routine is likely to be interrupted, and that interruption spells danger. In this way, scans and tests may evoke apprehension and suspense; ‘prognostic’ time, as Jain defines it, remains relevant. This temporality echoes the experience which Nancy K. Miller illustrates in her comic panel ‘Scanxiety’ (figure 1). Not a transplant recipient but a cancer survivor, Miller is aware of the rhythm of the calendar that structures her present and future in her ‘scan-to-scan life’, ‘living with the prospect of death, suspended between the unbearable present of treatment and the unknowable future, the fear of recurrence, triggered and retriggered by constant testing’ (Miller 2014, 221). Recipients’ post-transplant labour may involve an ambiguous state, an embodied enduring of strangeness, an as-yet-unfinishedness. For tissue transfer recipients, waiting does not end. The ‘for’ just changes.

Waiting need not necessarily be negative. The poet John Keats exalted the virtues and satisfaction of fostering a spirit of ‘negative capability’, tolerating ‘being in uncertainties, Mysteries, doubts, without any irritable reaching after fact & reason’, and mindfulness practices across many spiritual traditions have long valued suspended, attentive states (Chodron 2001; Keats 1958, 193–94). Waiting in illness contexts, too, may be more complex than merely distressing: it can be understood more capaciously as carrying ‘an assortment of expectations and hopes as well as frustrations’, as Sophie Day observes (Day 2016, 169). Waiting is also not necessarily lonely or even solitary: socialities of waiting can emerge, practices of ‘waiting with’ (Hage 2009; Salisbury 2020, 100). Nonetheless, waiting in apprehension and passivity may be an ordeal. This felt passivity can also be a challenge in illness more generally. Rita Charon suggests that:

When the doctor or nurse enters the room to do something … he or she remains within vectored time, that is, a state of time in which one event leads to another and can even be conceptualized as having caused it while the patient inhabits a timeless enduring. When the pediatrician John Lantos depicts the difference between being a patient and a doctor, he figures it in literary terms, suggesting that patients dwell in modernism’s focus on the interior while doctors enact the pre-modernist choice for acting, causing, derring-do. (Charon 2006, 44)

This passage describes the asymmetrical agency which may characterise the dynamic between patient and practitioner, particularly as represented within restitution stories of medical cure. Patients often have to be patient, and that patient patienthood blends passivity and agency in sometimes paradoxical ways. Deborah Steinberg describes how neoliberal patienthood idealises a certain faith in therapeutic trajectory: it involves a particular orientation in time, a compliance and active surrender in service of an imagined cured future (Steinberg 2015, 131–32).

Much work in medical humanities has sought to articulate and nurture patient agency within disempowering contexts (Frank 1995; Hawkins 1999; Kleinman 1988). Literature has been argued to be specifically of value insofar as it helps to give form to the distressing formlessness of illness time. Nadelhaft (2008), for example, describes literature ‘offer[ing] form, structure, and the illusion of dimension to what was out of control and without limit …. contain[ing] its immensity in a formal construction on the page’ (2008, 3). Such writing can be profoundly empowering. Yet it is useful to complement such language of ‘containing’ a self’s experience with other forms of critical noticing, such as scholarship which articulates ways that hegemonic conceptions of history and progress and cure may coexist with powerlessness and impasse (Ahmed 2004).

Waiting is always a material, embodied practice. It takes place in a wide range of institutional contexts, each riven with complex demands, legacies and financial imperatives, and it is affected by changing cultural assumptions about patient roles and about which lives merit which kinds of care (Jain and Kaufman 2011; Salisbury 2020, 100). Waiting is rich in diverse meanings for patients and practitioners and occurs within spaces with complex flows of force. It also means different things at different times. For centuries, western thought was influenced by Christian vigilance for the return of God and the end times, the eschaton (Koselleck 2004). ‘Church time’ arguably gave way to ‘merchant’s time’ in the middle ages, and the secularisation of time accelerated with the Enlightenment and the industrial revolution (Le Goff 1980). Dominant senses of futurity changed, as Reinhart Koselleck observes: ‘society was now perceived as accelerating toward an unknown and unknowable future, but within which was contained a hope of the desired utopian fulfillment’ (Koselleck 2004). Such strivings went alongside the emergence of the efforts to regularise and optimise life which Michel Foucault identifies as biopower, ‘bent on generating forces, making them grow, and ordering them’, operating on both a macrolevel and a microlevel and with its imperatives internalised by subjects (Foucault 1976). The industrial revolution inflected this futurity further, with the widespread influence of capitalist imperatives for productivity, labour on the clock.

The imperative to work, and to reproduce oneself for labour, has become further entrenched with contemporary neoliberal expectations about individual ‘resilience’ (Bracke 2016; Clarke et al. 2003; Neocleous 2013). The ‘waiting times’ of illness may be read as wasted time, failed time, in that it may be a period when a person might not fulfil the relentless rhythm of labour and reproduction of labour that characterises working life under capitalism. Cultural investment in striving for normative cure is inflected by neoliberal ideologies of productivity (Goodley 2005). Johanna Hedva (2016)warns, ‘The “well” person is the person well enough to go to work. The “sick” person is the one who can’t. What is so destructive about conceiving of wellness as the default, as the standard mode of existence, is that it *invents illness as temporary’*. Hours on waiting rooms chairs may not coexist well with the ‘chrononormative’ imperatives of the capitalist clock as Elizabeth Freeman describes it, ‘organiz[ing] individual human bodies toward maximum productivity … forms of temporal experience that seem natural to those whom they privilege’ (2010).

Illnesses like those which precede—and follow—transplantation may sit uneasily with medicine’s narratives of linear progress, as well as with wider cultural stories of how lives should flow. Jain describes the strange temporalities of ‘prognostic time’ with degenerative illness, noting that ‘living in prognosis severs the idea of a time line and usual ways one orients oneself in time: one’s age, generation, and stage in the assumed lifespan’ (Jain 2007). Long-term illness—such as pre-transplant deterioration and post-transplant care—may trouble assumptions of curative futurity.

Queer theory and crip theory offer ways to dwell critically with such transgressive temporalities. Queer theory offers multiple ways to think about time outside linear progress, finding alternative senses of time which may coexist within representations of such dominant arcs. Lee Edelman, for example, challenges reproductive assumptions that tend to underpin imagined futurities, and Jack Halberstam invites us to recognise the freedoms and potentials within ‘failure’ to mesh with conventional expectations (Edelman 2004; Halberstam 2011). Cultural assumptions about proper life stages are characterised by assumptions about heteronormativity, reproductivity, health and ability to work. Freeman develops Dana Luciano’s concept of a ‘chronobiopolitics’ to describe how institutions including the state ‘link properly temporalized bodies to narratives of movement and change’, in ‘teleological schemes of events or strategies for living such as marriage, accumulation of health and wealth for the future, reproduction, childrearing, and death’, and she finds ‘queer time’ evokes forms of temporality that may elude such hegemonic arcs:

I try to think against the dominant arrangement of time and history … in which historical narrative … organizes various temporal schemae into consequential sequence …. Instead, I track the ways that nonsequential forms of time (in the poem, unconsciousness, haunting, reverie, and the afterlife) can also fold subjects into structures of belonging and duration that may be invisible to the historicist eye. (Freeman 2010)

In different ways, then, ‘queering’ temporality means opening to the alternative futures beyond, for example, heteronormative reproductivity or predictable sequences. Such alternative forms of time can enable unexpected connection and community, pleasures flowering in temporal interstices. Yet ‘queer time’ may also be understood as wistful, melancholic, static and even deathly, as Heather Love shows, sometimes framed pejoratively as ‘backward’, immature, failing to fulfil evolutionary expectations (2007). If a culture figures futurity as the multiplication of life through reproduction, then refusal of that trajectory would seem deathly by the yardstick of that culture and, by extension, can be read as symbolic of a rejection of life. Edelman, drawing on Lacanian hermeneutics, notes: ‘the death drive names what the queer, in the order of the social, is called forth to figure: the negativity opposed to every form of social viability’ (2004). While not all queer theorists read queerness as negation, many comment on queerness as culturally associated with deathliness, unnaturalness and refusal. Love and Freeman, for example, recognise senses of community that entangle awareness of death and impasse, and Halberstam acknowledges ‘history of representations of homosexuality as loss and death from Proust to Radclyffe Hall’ (2011).

Within disability studies, too, ‘crip theory’, itself indebted to queer theory, identifies alternative temporalities to particular idealisations of productive progress. Disabled experience can sit particularly uneasily with the way such progress arcs assume ‘curative time’, in which bodies are healed and normalised within a culture’s expectations. In an effort to carve out imaginative space for alternatives to such temporality, Alison Kafer adapts the concept of ‘crip time’—a wry, in-group term long affectionately used within communities of people living with disability, to denote the way things may take longer for us when dealing with maladaptive environments or other challenges. Kafer broadens the term to encompass alternatives to curative temporality:

Futurity has often been framed in curative terms, a time frame that casts disabled people (as) out of time, or as obstacles to the arc of progress. In our disabled state, we are not part of the dominant narratives of progress, but once rehabilitated, normalised, and hopefully cured, we play a starring role: the sign of progress, the proof of development. (Kafer 2013, 28)

Similarly, Ellen Samuels describes crip time as ‘time travel’, in that:

Disability and illness have the power to extract us from linear, progressive time with its normative life stages and cast us into a wormhole of backward and forward acceleration, jerky stops and starts, tedious intervals and abrupt endings …. The medical language of illness tries to reimpose the linear, speaking in terms of the chronic, the progressive, and the terminal, of relapses and stages. But we who occupy the bodies of crip time know that we are never linear. (Samuels 2017, para 5)

In cultures which assume worthy lives are those which are conventionally productive in a capitalist sense, ‘crip time’ may at times be used as a slur. Adapted by Kafer et al., the term is informed by awareness of social cruelties, maladaptive environments and the ways that disablism exacerbates suffering, prolongs time and effort to do things and curtails futures. Yet despite these bleak dimensions, the concept of ‘crip time’ does not equate with inevitable despair, but rather conveys ambiguity and creativity, alert to the perils of structural conditions and normative assumptions even while seeking to imagine and articulate alternatives.

Transplantation memoir, fiction or film-making may confirm progress narratives of triumphant medical advance and hegemonic conceptions of normative reproductive futurity in, for example, memoir, media reports, fiction or films, which idealise intrafamily donation as confirming existing familial bonds, while resisting more unconventional intimacies that might develop between, for example, recipient and donor kin (Sharp 2006; McCormack 2016b, 57). The concept of enfolded temporalities, coexisting within yet counter to dominant arcs, may be valuable in approaching illness narrative, particularly works which do ostensibly describe medical success. Queer and crip scholarship offers language for noticing exactly such hidden adjuncts to dominant arcs of story, hegemonic futurities and historicities. For Freeman, identifying these other kinds of time can be about pleasure, delight and community, available within even hostile contexts and pasts. By contrast, this article identifies sideways, hidden, slant temporalities to a different end, carving out a way of noticing ambiguous figures within illness memoir in which heroic secular ‘miracle’ may simultaneously convey strain, uncertainty and consciousness of death. While not playful or pleasurable, these are nonetheless sideways, illicit, non-normative temporalities, complicating the restitution arc.

Elsewhere I consider how an early cinematic fantasy of tissue transfer, Franju (1960)’s *Les yeux sans visage* (1960), shows progressive rejection of skin graft on screen, unfolding over time. The decay of the skin is accompanied by a dramatisation of the postsurgical melancholy of the transfer recipient, Christiane, afflicted by inevitable rejection in the absence of effective immunosuppression:

the film’s fantasticality, its dream-like evocation of post-transplant suspense and melancholy, is anachronistically valuable now in expanding our rhetorics of potential post-transplant temporalities and subjectivities: the intuition of the body made strange, the sense of a restored ‘intactness’ that feels not one’s own, and the need to watch and fear for clues of graft failure. While the film may be fantastical, these affects are not. (Wasson 2020, 147).

The memoirs that I examine in this present article describe medically successful tissue transfer, unlike the failed transfer depicted in the fantastical *Les yeux*, but these works, too, feature productive ambiguities, showing how triumph and estrangement may coincide.

## Secular revision of the Lazarus figure in transplant memoir

I will now examine two memoirs by transplant recipients which grapple with its odd temporalities. Other memoirs also convey the unfinishedness of transplant, most famously Jean-Luc Nancy’s ‘L’Intrus’ (2000) and Francisco Varela’s ‘Intimate distances’ (2001). Nancy describes aftermath as a ‘gaping open’, ‘*béance’,* ‘an opening through which passes a stream of unremitting strangeness: the immuno-depressive medication, and others’ (Nancy 2002, 10–13). Varela, in turn, describes the aftermath in terms of his body being occupied as though *by* death as itself a force, a presence: ‘that night when death travelled through my open body is to remain indelibly’ (Varela 2001). The memoirs I discuss next complement such representation by offering a different kind of figuration: a secular version of the Biblical figure of Lazarus, whom both writers make into an emblem of simultaneous resurrection and degradation.

The waiting in these texts has three elements: first, it occurs within non-curative time, and—related—this waiting is effort, painful, often exhausting, a long-term labour. Second, this waiting is *social*, a waiting-with others. Third, this waiting is informed by an awareness of death: death is part of the waiting ‘for’, and awareness of death somehow infuses all other aspects of experience. These three dimensions of waiting invite connections with crip theory’s recognition of eluding curative time, and the intuition of a sociality within waiting that emerges within both crip and queer writing, including intuitions of connections occurring within and across death.

Robert Pensack’s memoir *Raising Lazarus*, cowritten with Dwight Williams, describes Pensack’s lifelong struggle with what was known at the time as idiopathic hypertrophic subaortic stenosis, now called hypertrophic cardiomyopathy (Pensack and Williams 1994). In multiple ways, this book foregrounds an arc of triumphant cure. The memoir celebrates heroic medicine and a protagonist invested in their own resilience: the book’s epigraph is from Virgil’s Aeneid: “I sing of warfare, and a man at war”, and the metaphor of illness as battle recurs throughout (107, 142, 189). The foreword by famous surgeon Thomas Starzl positions Pensack’s experience firmly within a recovery narrative mediated by science, describing it as a ‘grand adventure story’ of a man healed by medical advances (Starzl 1994, xii). Starzl goes further, explicitly posing a parallel between Pensack’s story and the story of the coming to fruition of transplantation as a feasible medical intervention: ‘This book also is an epic of progress in the field of transplantation which dawned in 1962 at almost the same time that Bob Pensack’s medical diagnosis was made, and reached full bloom in time to save him thirty years later’ (xi).

The memoir is unusual in that it combines those two forms of agency: the resilient patient and the heroic doctor. Starzl’s foreword foregrounds Pensack’s own medical and scientific agency: ‘Remarkably, [Pensack] played a role in this revolution by helping to produce one of the antirejection drugs with which he was treated at the time of his greatest need’ (Starzl 1994, xii). Starzl hails Pensack as himself a medical agent, both a beneficiary and an agent of cure. Pensack’s own narration continues this theme. It opens with him collapsing on the floor of his kitchen while waiting for a heart transplant. Paramedics gather around him, and we hear that ‘as a circle of faces forms above me I tell them I’m a doctor, that this is not a typical heart attack, that I know exactly what’s going on’ (Pensack and Williams 1994, 4–5). His medical expertise even inflects his reaction to the prospect of imminent transplant surgery, taming his panic by invoking his own medical professionalism—“As a medical student, I learned that the first pulse you take upon entering a medical emergency should be your own”—and as he washes himself before his surgery, he ‘scrub[s] with the obsessive care of a surgeon, moving the washcloth from skull to toe’ (8–12). He is triumphant when he starts to decipher his own cardiac illness: “I am a spy in the house of my heart” (100). When his condition deteriorates to a stage that he needs a pacemaker, Pensack researches pacemakers and selects the most advanced, and watches when it is installed under local anaesthetic (149, 155–57). He also successfully diagnoses himself psychiatrically with postsurgical trauma (166–67). After the transplant, too, Pensack continues to actively shape his own treatment: he contacts Starzl to ask for the immunosuppressant FK-509, not yet publicly released, and Starzl comments on ‘The theme of “physician heal thyself”’ (xii). Pensack is also involved, although tangentially, with experimental medicine: early in adulthood, he works as a medical researcher injecting human lymphocytes into animals to manufacture antilymphocyte globulin (ALG), an immunosuppressant which saves him 20 years later. Pensack’s agency is repeatedly tied to the extent to which he is a medical practitioner, a resilient patient and a pathbreaking agent operating within curative time. Throughout, Pensack wants to master his body: “I want to know it all, master the science … become intimate with its secret workings” (179).

Yet these triumphant temporalities of progress and vectored agency are interwoven with experiences of time which are simultaneously less linear and less centred on a knowing, medical agent. Other experiences of time emerge: the temporalities of ominous portent, of traumatic flashback, of psychotic delirium in an eternal present, and a daily routine touched with the trope of haunting. Each such passage troubles, from within, the narrative of heroic healing. Initially, these hints of disruptive temporality are readily recuperated back into medical triumphalism. Early in the narration, when driving to hospital for a potential transplant, Pensack feels apprehensive as his wife phones friends to advise of the imminent possible surgery:

gradually the receiver fills with static as we progress out of range. The inability to remain in contact with the world, the suddenly eerie silence, filled the car with a sense of foreboding. Here we are cruising beneath the waxing moon that stands over a vast shadowy forest, the green and orange fluorescent lights illuminating our faces in a surreal cast, on our way to a heart transplant. But the portent is short-lived. In spite of flying in the face of mortality, lying myself down beneath the knife commonly known as heroic medicine … I grow optimistic …. I'm ecstatic. I want this heart. (9)

Confidence continues to characterise his expectations of his own healing, and as described above, the narration generally presents surgeries as highly empowering, in which he is positioned as an agent rather than a passive participant. Yet there are exceptions, and one of the most striking occurs during a heart catheterisation, when he is treated as merely a normal patient. He senses something is wrong but his attempts to communicate his concerns are dismissed with platitudes. His panic mounts:

I feel the Swan [Swan-Ganz catheter] floating down my neck, and with it comes the overwhelming grip of claustrophobia. Suddenly something feels wrong. An eerie movement high up in my chest …. The potential for devastating infection, heart attack, arrythmia, or puncturing the heart wall is always there. The procedure is no less bizarre and seemingly barbaric than something dreamt by a diseased mind, the mind of Dr Frankenstein, Dr Moreau. Yet my tentative future lies within the sleepless walls of this hospital, in the cold hands of masked surgeons. I am merely a purveyor of nameless static rage. (212–13)

Pensack uses explicitly Gothic intertextualities, in line with a long tradition of life writing and scientific writing informed by that literary mode (Davison 2017; Kennedy 2004; Punter 1998; Servitje and Vint 2016; Smith 2004; Smith 2016; Stephanou 2014; Talairach-Vielmas 2009; Wasson 2015). In this lurid scene, he invokes the literary figures of Frankenstein and Moreau to convey his sense of his own diminished agency, and the passage shows helplessness and a specific, alternative sense of time. He waits in suspense while intuiting a future governed by curse and fatality, a ‘tentative future’ within ‘sleepless walls’ and under ‘cold hands’, helpless and confined.

A similar chilling futurity emerges during his bouts of postsurgical psychological distress, after both his initial pacemaker insertion and his heart transplant. During both these experiences, ‘vectored’ time gives way to an endless and menacing present. During surgery to narrow the muscle constricting his heart, his heart’s electrical function is permanently impaired and a pacemaker is implanted. After this surgery, Pensack experiences profound depersonalisation, agoraphobia and traumatic flashback, feeling “at the mercy of a chaotic universe with no order, no security… All I know is that I am naked and alive”; “as I sit in the vacuum of darkness and silence… I think of those ancient photographs of dead relatives on my father’s nightstand. Their eyes gazed back almost expressionless, as though they were remembering us, not us remembering them. The haunted legacy” (122, 132–33). Here, postsurgical trauma disrupts a sense of linear medical progress and a personal sense of history and agency. Times flows in strange ways, the dead remembering the living-who-are-yet-to-live. Intense psychological dysfunction also follows the transplant surgery itself, when the unusually long surgical procedure and his reaction to prednisone triggers ICU psychosis:

my body lies still, as though shattered by what it has seen. All about me is a jangled music of beeping woven into a liquid light, tainting all colour that of the laboratory of Dr Moreau. This scene isn't so futuristic as it is macabre. From out of my chest extend tubes that drain fluid from the hollowed pocket holding my heart as it quivers …. The entire body holds the general yet acute expression of shock in its utter stillness… I should be dead and the body knows this …. I grow insane, I have no concept of time …. It is a private nightmare, a wilderness of mirrors. (258–59, 261, 265)

The definite article—‘the body’—shows his estrangement from his own body. This fractured state is in opposition to the framing of heroic medical agency that dominates the rest of the memoir, yet literally coexists with one of the cornerstones of that version of that empowering story, for it is now, in this state, that he needs the treatment with the ALG which he helped manufacture decades earlier (258). Despite both Starzl and Pensack foregrounding his earlier contribution to the development of ALG, in the moment that when he actually receives it, the temporality of heroic cure entangles with timeless madness and suffering.

Pensack’s meshing of the modes of miracle and chronic crystallises in his use of the Lazarus image. Pensack uses the word ‘miracle’ often, such as in his 2005 afterword: twelve and a half years after the transplant, Pensack declares, “I continue to be one of the true miracle stories of organ transplantation” (319). The very title of his memoir, too, invokes miracle. The Biblical story of Lazarus is brief, but telling. Four days after Lazarus had been entombed: ‘Jesus shouted, “Lazarus, come out!” And the dead man came out, his hands and feet bound in graveclothes, his face wrapped in a headcloth’ (John 11:1–44, NRSV). As healing miracles go, however, the Lazarus story is one that confronts readers with the materiality of death. Fundamentally, the Lazarus story is not transcendent, but immanent; unlike the Ascension story of Jesus’s mother Mary, for example, Lazarus stays very much on the earth, with a vivid memory of death.

Similarly, while at first glance Pensack invokes the story to frame transplantation as miracle, closer scrutiny shows his own resurrection as entangled with degradation and weary, ongoing struggle. Both Pensack and McCann embrace the ambiguity of the Lazarus figure. Pensack unflinchingly shows the devastation attendant on recovery from the surgery—“I should be dead and the body knows this”—and after the intensity of surgical aftermath, daily maintenance requires effort: “to perform simple tasks without the preoccupation of this invisible illness haunting every motion is gone forever…… I am not all better now as everyone, myself included, would like to believe” (308). The chronic everyday incurs the language of haunting, and he is continually vividly aware of the closeness of death in ways that many other people have the luxury of forgetting. Lazarus, too, emerged still in his graveclothes. Pensack’s memoir ultimately invokes the temporality of ‘miracle’ not in order to convey the heroic moment of transplantation surgery, but to convey the ‘shattering nature of recovery’ (273), the difficulty of enduring the aftermath of miracle. The Lazarus figure conveys both the glory of medical agents—with whom Pensack himself identifies—as well as the decaying, vulnerable subject who is raised but still wrapped in the cloths of death.

McCann’s essay is also a story of successful transplant, in his case a liver transfer in response to hepatitis. He is aware of the astonishing feat that surgeons have wrought for him: “The liver of a dead person was placed inside me so I might live again” (101). The transplantation is successful, but McCann shuns the idea of writing a triumphant story. In interview, he says that he never wanted to write about his experience *as ‘*the story of a liver transplant’:

It’s never been a medical adventure to me. To write the story that way, to me, would be as appalling as writing a case history …. The basic narrative of transplantation—what people believe—is: you are sick, you get an organ by the grace of God or whatever, and now you’re well. But transplantation is not a restoration narrative. It’s not a narrative of a break and then a resumption. Your narrative is altered, utterly, from the experience. You go on immunosuppressive drugs, which are highly toxic, for the rest of your life. You trade in your imminent death for—as time goes on—a series of simultaneously more manageable and more life-threatening conditions. (Porter and McCann 2010, 117)

To avoid the too-neat story, McCann shows time as non-linear even before surgery. He overhears a nurse saying that many teenage donors die from suicide, and McCann is shaken by that knowledge. He lies down in a park to distract himself:

I was lying on the unmowed grass … pretending that my whole life consisted of just one word: *sunny, sunny …*. But it didn't work. My donor had begun to claim me, or so it seemed; I felt as if he'd somehow been constructing himself inside me without my knowledge as I was dying, though he was [sic] still alive and waiting for nothing unforeseen. (McCann 2002, 104–5)

Having destabilised conventional senses of time, it is not surprising when McCann’s narration proceeds to show the post-transplant time as far from a restored state. Like Pensack, McCann uses the image of Lazarus less to foreground surgical miracle than to foreground post-surgical suffering:

what did Lazarus want after he stumbled from the cave, tied hand and foot with grave clothes, his face bound about with a napkin… I want to complete what I’ve written here—these fragments, these sticky residues of trauma—by adding just one more line before the words the end: ‘It’s a miracle’. …. But what happens after the miracle? What happens after the blinding light of change withdraws and the things of the earth resumed their shadows? (107)

What happens is effort, routine, anticipation and dread. In McCann’s memoir, as in Pensack’s, the primary function of the Lazarus metaphor is to convey the extreme effort that must follow resurrection:

Each morning and evening I monitor myself for organ rejection, as I’ll do for the rest of my life: blood pressure, temperature, weight. I go to the clinic for blood draws; I await faxes detailing test results.

Here is what happens after the resurrection:

Your body hurts, because it’s hard to come to life again after lying so long in a grave, but you set goals and you labour to meet them …. You learn your medications, you learn to pack your wounds with sterile gauze; you learn to piss into a bottle and shit into a pan. It’s work, preparing yourself for sunlight. (109)

## Waiting in company; waiting and death

This work is not done alone. In addition to using the Lazarus trope to convey the labour of after-the-miracle, both Pensack and McCann show touching, sad and awkward socialities, and show the ongoing waiting as occurring alongside an abiding, intense awareness of death. These elements, too, are part of the Lazarus story. The Biblical story offers two details of Lazarus’s aftermath: he returns wrapped in graveclothes, and returns to his relatives’ arms. After experiencing both glorious miracle and squalid decay, he remains embodied and closely enmeshed in relationships with other people. Both Pensack’s and McCann’s texts, too, show sociality in the aftermath of miracle, and both also show that ongoing life as inextricably entangled with consciousness of death.

Although the image of ‘waiting’ might connote a solitary, lonely figure, waiting is fundamentally a social practice. Ghassan Hage observes that ‘Waiting indicates that we are engaged in, and have expectations from, life’ (2009). Waiting is a waiting *for*, and as Laura Salisbury has shown, can readily become a waiting *with—*both prepositions show the waiter situated within relationship with others (2020, 100). In this regard, Pensack talks about his relationship with his wife, children, colleagues and friends, as well as other heart patients he meets and supports through their own medical experiences. As he waits for cardiac surgery with others also enduring the same condition, they share ‘war stories’: ‘There is no need for shame or fear, only the quiet satisfaction of camaraderie, the brand-new sense of recognizing yourself in others’ (107). Both Pensack and McCann also handle changes in relationships with loved ones affected by their own deterioration and surgeries. Most poignantly, both texts come to terms with the impossible-yet-vivid sociality of their relationship with the deceased donor, feeling guilt and tenderness about the tissue taken into themselves. This, too, is reported with oddities of time. Pensack dreams of being at a traffic accident where the donor died: “As I move toward the scene tears gush over my cheeks” (301). A police officer at the scene becomes suspicious that Pensack was responsible for the crash. When he wakes, he feels strange, the dream ‘haunt[ing] the day like the presence of a friendly ghost. There is an air that something is about to happen’ (301). Soon, he discovers that his wife had a dream simultaneously in which she and he were on trial for murdering the donor. The sharing of the dreams triggers a release, a rush of sadness, grief and love. McCann, in turn, touches his own body at night with guilt, grief and kindness:

I placed my hand over what seemed to be still her liver, not mine, and slowly massaged the right side of my body—a broken reliquary with a piece of flesh inside—all the way from my hip to the bottom of my rib cage. “It’s okay, it’s okay”, I whispered over and over, as if I were attempting to quiet a troubled spirit not my own. (106)

This attitude of parent-like kindness has been reported by other transplant recipients (Varela 2001). In such gestures, McCann enacts tenderness to someone dead and someone damaged: the donor and himself. Together, they endure aftermath, and during the monitoring they find McCann’s hepatitis is inevitably destroying the new liver: “The hepatitis goes on, the doctor tells me. The transplant doesn’t cure it. It gives the virus a new liver to infect and feast upon. (*Dear donor, forgive me, I can’t save your life*)” (107). As mentioned earlier, scholarship on queer temporality has long reiterated the value of complex, difficult and impossible figurations of connection with the dead (Freccero 2005; Freeman 2010; Love 2007). Those waited ‘with’ may be others enduring the same illness challenges, loved ones who care, or, at its strangest, the dead whose tissue has been incorporated.

Lazarus’s Biblical story does not flinch from the squalor of death. Lazarus’s sister warns that the smell of the decayed body will be terrible if the gravestone is rolled aside, since her brother has been buried for 4 days. McCann and Pensack, too, confront the way the ongoing life of postsurgery is an oddly deathly life. Pensack says often that he feels kinship with veterans and survivors of war and atrocity, because “What I have come to know with terrifying intimacy isn’t something that can be shared around dinner tables. The traumatised Vietnam veteran, the Holocaust survivor with flat, distant eyes—these are my people, those with whom I feel kinship… Through a lifetime I have been in the process of dying” (6–7). McCann admits, “you can’t forget how it felt to lie in the close darkness of that grave; you can't forget the acrid smell of the earth or the stink of the mouldering grave clothes …. The gift of life is saturated with the gift of death” (109). Both Pensack and McCann use the Lazarus myth to convey the profound sense of death now inhabiting awareness of life.

Both memoirs, then, use the story of Lazarus as a structuring trope, to entangle two different temporalities across one image: the miraculous intervention moment of Lazarus being raised/the intervention of the transplant surgeon, and the grinding, bewildering duration of aftermath, of chronic illness and ongoing management. When used in a religious context, the Lazarus story can be seen as combining both the *eschaton* of the end times with the immanence of ongoing life: Lazarus stays on the human plane. The story is eschatological without transcendence. Figurations which can combine the exalted temporality of miraculous moment, and the ambiguous, even squalid duration of aftermath, are worth excavating: they may help meet the representational challenges of transplant time.

## Conclusion

This discussion has sought to resist reducing the ‘reality’ of post-transplant experience to either single healing event or bleak extended duration. Within these two memoirs, the figure of Lazarus comes to serve, in different ways, to entangle these two kinds of time. Alongside this image, I wish to invoke another concept from affect theory, which speaks to the productive ambiguity of these states: Sianne Ngai’s notion of ‘stuplimity’. Blending stupid and sublime, the coinage conveys ‘the experience of boredom increasingly intertwined with contemporary experiences of aesthetic awe …. [T]he initial experience of being aesthetically overwhelmed involv[ing] … not terror or pain … *but something much closer to an ordinary fatigue’* (Ngai 2007, 8, 270, emphasis in original). This fatigue stems from the ongoing labour to endure, experience, witness the conceptually overwhelming. The term is relevant in describing the textures of pre-transplant and post-transplant experience and the workings of time. Her term conveys a state of being both desensitised and buffeted by wonders—the dreariness of the marvellous.

In that spirit, the waiting of these texts has three dimensions, and it is possible that this commentary may be of value, too, in approaching some other memoirs of heroic medicine. The waiting in these memoirs occurs within non-curative time, and the corollary of this is that waiting is effort, often exhausting, a long-term labour. It is social, a waiting-with others. Finally, it is informed by an awareness of death: not only is death part of the waiting 'for', but death also infuses all other aspects of experience. In all these ways, such waiting connects with crip and queer efforts to recognise the validity of unexpected, sideways, rogue ways of approaching time beyond linear progress arc. This article has suggested that there is value in finding additional literary figures and narrative devices that can express such paradox, and critical practices which can describe the multilayering of narrative arcs and hidden temporalities. Such multiple times may be represented simultaneously through condensed symbolic figuration. The Lazarus image in these works blends the image of miraculous surgery with the language of chronicity, toil and the unhealable. I am reaching for a critical practice to recognise subtle entanglements of medicalised time, ways they may meet on a page and even within a single figure: a figure waiting, a figure strange, living on with others and living on with death.

## Data Availability

Data sharing not applicable as no datasets generated and/or analysed for this study.
